# The Oogenic Germline Starvation Response in *C. elegans*

**DOI:** 10.1371/journal.pone.0028074

**Published:** 2011-12-02

**Authors:** Hannah S. Seidel, Judith Kimble

**Affiliations:** 1 Department of Biochemistry, University of Wisconsin-Madison, Madison, Wisconsin, United States of America; 2 Howard Hughes Medical Institute, Madison, Wisconsin, United States of America; Brown University, United States of America

## Abstract

Many animals alter their reproductive strategies in response to environmental stress. Here we have investigated how L4 hermaphrodites of *Caenorhabditis elegans* respond to starvation. To induce starvation, we removed food at 2 h intervals from very early- to very late-stage L4 animals. The starved L4s molted into adulthood, initiated oogenesis, and began producing embryos; however, all three processes were severely delayed, and embryo viability was reduced. Most animals died via ‘bagging,’ because egg-laying was inhibited, and embryos hatched in utero, consuming their parent hermaphrodites from within. Some animals, however, avoided bagging and survived long term. Long-term survival did not rely on embryonic arrest but instead upon the failure of some animals to produce viable progeny during starvation. Regardless of the bagging fate, starved animals showed two major changes in germline morphology: All oogenic germlines were dramatically reduced in size, and these germlines formed only a single oocyte at a time, separated from the remainder of the germline by a tight constriction. Both changes in germline morphology were reversible: Upon re-feeding, the shrunken germlines regenerated, and multiple oocytes formed concurrently. The capacity for germline regeneration upon re-feeding was not limited to the small subset of animals that normally survive starvation: When bagging was prevented ectopically by *par-2* RNAi, virtually all germlines still regenerated. In addition, germline shrinkage strongly correlated with oogenesis, suggesting that during starvation, germline shrinkage may provide material for oocyte production. Finally, germline shrinkage and regeneration did not depend upon crowding. Our study confirms previous findings that starvation uncouples germ cell proliferation from germline stem cell maintenance. Our study also suggests that when nutrients are limited, hermaphrodites scavenge material from their germlines to reproduce. We discuss our findings in light of the recently proposed state of dormancy, termed Adult Reproductive Diapause.

## Introduction

Many animals alter their reproductive strategies in response to environmental stress [1], [2], [3], [4], [5]. In the free-living roundworm *Caenorhabditis elegans*, adult hermaphrodites respond to food deprivation by retaining their embryos in utero [6], [7], [8]. Embryos then hatch within the body of the parent hermaphrodite and consume the parent in a process known as ‘bagging.’ Bagging invariably kills the parent, but it may provide a nutritional advantage to the progeny: During starvation, progeny hatched inside a bagging animal are able to reach the stress-resistant dauer stage more often than progeny hatched outside [9], [10]; when the parent's body is divided among fewer progeny, dauer formation is increased [9], [10]. Thus, it has been suggested that bagging is an ecologically relevant strategy maximizing offspring survival during starvation [9], [10].

Upon starvation from the L4 stage, some *C. elegans* hermaphrodites do not bag as adults but instead survive [11]. The non-bagging animals were proposed to have entered a state of dormancy, termed Adult Reproductive Diapause [11]. In support of this diapause state, it was proposed that animals avoid the bagging fate because their embryos enter a state of embryonic arrest or slowed development, in which these embryos remain stalled in the first half of embryogenesis for up to five days [11]. Other proposed features of Adult Reproductive Diapause include (i) germline shrinkage during starvation; (ii) capacity for germline regeneration upon re-feeding; and (iii) a requirement for crowding [11].

We have starved hermaphrodites from the L4 stage and quantified the fraction of animals that survive the first ten days of starvation. We have tested whether embryos produced during starvation remain in early embryogenesis for more than ∼24 h. We have also tested whether germline shrinkage and the capacity for germline regeneration are unique to non-bagging animals. Finally, we have tested whether the starvation response depends upon crowding. Our results challenge the existence of a specialized program of dormancy that permits animals to escape the bagging fate. Instead, our results suggest that all L4 hermaphrodites respond to starvation equivalently, with the death or survival of each animal depending on its ability to produce viable progeny during starvation.

## Results

### Effect of starvation on survival

**Figure 1:**
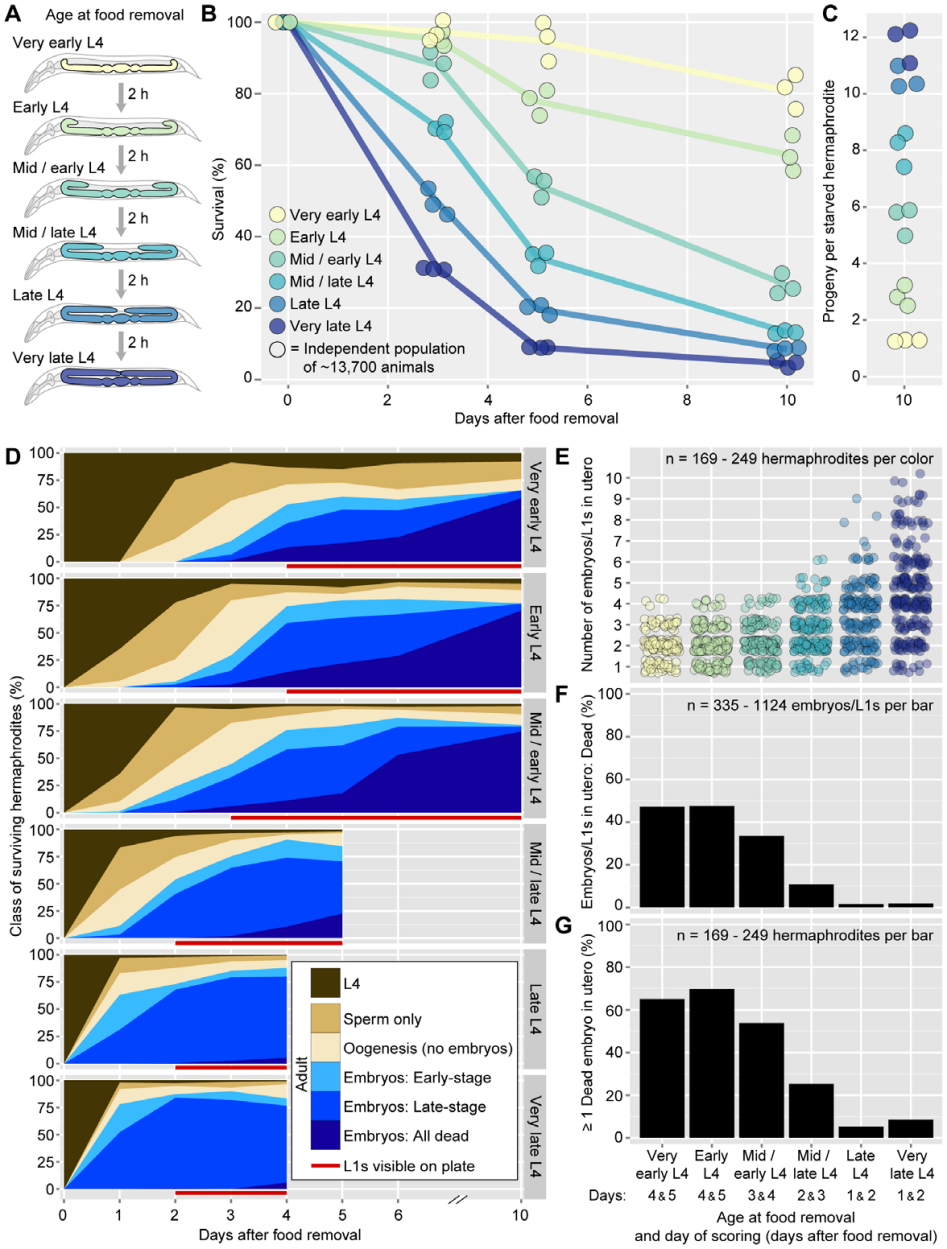
Effect of starvation on survival and embryo production. (A) Developmental stages at which starvation was initiated, shown according to the extent of gonad growth. (B) Survival curves for populations starved from ‘Very early’ to ‘Very late’ L4, as defined in (A). (C) Total larval progeny, per hermaphrodite, on day 10 of starvation, calculated as the final number of larval progeny relative to the initial number of starved hermaphrodites. In (B–C), circles represent replicate populations, each containing ∼13,700 animals at the onset of starvation. Curves in (B) connect averages of each set of replicates. (D) Classes of surviving hermaphrodites in populations starved from ‘Very early’ to ‘Very late’ L4. For each population, on each day, *n* = 149–152 animals. Classifications are as follows. ‘L4’ = L4 larvae. ‘Sperm only’ = Adult that has not yet begun oogenesis. ‘Oogenesis (no embryos)’ = Adult containing oocyte material in at least one germline arm and no embryos or L1s. (Oocyte material was defined as granular cytoplasm characteristic of oocytes.) ‘Embryos: Early-stage’ = Adult containing at least one viable-looking, early-stage embryo and no late-stage embryos or hatched L1s. ‘Embryos: Late-stage’ = Adult containing at least one hatched L1 or viable-looking, late-stage embryo. ‘Embryos: All dead’ = Adult containing at least one dead embryo and no viable-looking embryos or hatched L1s. Red lines indicate the appearance of L1s outside the bodies of parent hermaphrodites. Because starvation inhibits egg-laying, and starved populations never contained laid embryos, the appearance of L1s acts as an indicator that some animals have already died via bagging. Once L1s appear, samples become biased towards animals that have produced fewer viable embryos prior to that day of sampling. (E) Number of embryos or L1s in utero for animals containing at least one embryo or L1. Circles represent individual hermaphrodites. (F) Percent of fertilized progeny in utero that have resulted in dead embryos (dead embryos/total embryos and L1s). (G) Percent of embryo/L1-containing animals that contain at least one dead embryo. (E–G) For each ‘Age at food removal,’ data derive from the first two days in which at least one third of the population contained embryos or L1s. The full dataset is shown in Figure S1.

We have investigated how L4 hermaphrodites respond to starvation. To initiate starvation, we removed food at 2 h intervals from early- to late-stage L4 animals (Figure 1A). We refer to these stages as ‘Very early’ L4, ‘Early’ L4, ‘Mid/early’ L4, ‘Mid/late’ L4, ‘Late’ L4, and ‘Very late’ L4. Food was removed by washing animals repeatedly until no bacteria were visible in the resulting supernatant. After food removal, animals were plated at a density of at least 10,000 animals per 10 cm plate. This procedure removed the vast majority of food, as judged by the absence of bacterial growth on the resulting plates.

We monitored starved populations throughout the first ten days of starvation. In all populations, animals either survived or died via facultative matricide: Food removal inhibits egg-laying [6], [7], [8], such that embryos hatch within the body of the parent hermaphrodite and kill the parent in a process known as ‘bagging.’ Deaths from other causes were rarely, if ever, observed.

To estimate the fraction of animals surviving versus bagging, we estimated the fraction of animals remaining alive on days 3, 5, and 10 of starvation. Animals starved from early L4 survived more often (and bagged less often) than animals starved from late L4. For example, by day 10 of starvation, 76%–85% of animals starved from ‘Very early’ L4 remained alive, compared with only 3–5% of animals starved from ‘Very late’ L4 (*n* = 3 populations of ∼13,700 animals) (Figure 1B). Consistent with this difference, populations starved from early L4 produced fewer larval progeny by day 10 of starvation than populations starved from late L4 (Figure 1C). Thus, although all animals either survive the first 10 days of starvation or die via bagging, the fraction of animals adopting each fate depends upon the age at which food removal occurs.

### Effect of starvation on embryo production

To understand the differing rates of surviving versus bagging for animals starved from early versus late L4 (Figure 1B), we sampled surviving animals from starved populations on days 1 to 6 and day 10 of starvation. In each sample, we recorded the fraction of animals that had (i) molted into adulthood, (ii) begun oogenesis, and (iii) produced at least one embryo (Figure 1D). In animals containing embryos or L1s, we recorded the total number of embryos or L1s present and the developmental stage of each embryo. For all animals that had begun oogenesis, we estimated the approximate volume of oocyte material in each germline arm; this record serves as a crude metric of progress towards oocyte completion and the next ovulation.

From this dataset, we identified three major differences between animals starved from early versus late L4. First, in animals starved from early L4, three processes were severely delayed: (i) the molt into adulthood; (ii) the onset of oogenesis; and (iii) the onset of embryo production (Figure 1D). For example, in populations starved from ‘Very early’ L4, on day 1 of starvation, all animals remained at the L4 stage (*n* = 150); on day 2, 75% (*n* = 150) of animals had molted into adulthood, but only 27% had begun oogenesis; embryo production did not begin until day 3, and even on day 3, only 19% (n = 151) of animals contained at least one embryo (Figure 1D). By contrast, in populations starved from ‘Very late’ L4, on day 1 of starvation, 78% (*n* = 151) of animals contained at least one embryo; moreover, 12% of animals were already bagging (as defined by one or more hatched L1 in utero) (Figure 1D). Animals starved at intermediate stages formed a continuum between these extremes (Figure 1D). Thus, starvation from early L4 delays the molt into adulthood, the onset of oogenesis, and the onset of embryo production, and each 2 h decrease in the age at food removal causes this delay to be incrementally more severe.

Second, animals starved from early L4 produced fewer total embryos during starvation than animals starved from late L4: Animals starved from ‘Very early’ or ‘Early’ L4 typically contained three embryos/L1s or fewer, whereas animals starved from ‘Very late’ L4 often contained five embryos/L1s or more (Figure 1E, Figure S1A). Third, animals starved from early L4 showed higher rates of embryo lethality than animals starved from late L4 (Figure 1F, Figure S1B). For example, in populations starved from ‘Very early’ or ‘Early’ L4, on the first two days in which at least one-third of animals contained embryos or L1s, 47%–48% (*n* = 275–471) of embryos were already dead (Figure 1F, Figure S1B); likewise, 65%–70% (*n* = 169–231) of embryo/L1-containing animals contained at least one dead embryo (Figure 1G, Figure S1C). By contrast, in populations starved from ‘Late’ or ‘Very late’ L4, the comparable rates of dead embryos were less than 2% (*n* = 704–1124) (Figure 1F, Figure S1B). Thus, relative to animals starved from late L4, animals starved from early L4 produce fewer total embryos during starvation, and fewer of those embryos are viable.

Together, these differences in embryo viability and the timing of embryo production explain the differing rates of surviving versus bagging for animals starved from early versus late L4. During the initial days of starvation, animals starved from early L4 cannot bag because they have not yet begun embryo production. Once embryo production begins, animals starved from early L4 often still fail to bag because they fail to produce even one viable progeny.

Finally, the delay in embryo production for animals starved from early L4 seemed to reflect a prolonged time required for oocyte completion. During days 1 to 6 of starvation, in oogenic (but embryo-less) animals starved from ‘Very early’ to ‘Mid/early’ L4, 78.2% (*n* = 856) of germlines contained oocyte material totaling less than half the volume of a mature oocyte. The infrequency of mature-sized oocytes in these animals suggests that their germlines were not withholding oocytes prior to ovulation – but instead, that oocyte completion was delayed.

In addition, despite embryo production being delayed, embryo production did not continue throughout starvation. By day 10, ovulations largely ceased, as evidenced by the near absence of viable-looking embryos (Figure S1B) and the complete absence of mature-sized oocytes (*n* = 771 oogenic germlines). Therefore, on day 10 of starvation, the animals remaining alive had either never begun embryo production in the first place or had produced only dead embryos.

### Tracking single animals during starvation

We considered two explanations for the observation that starved populations contained non-bagging animals with one or two viable-looking, early-stage embryos up to five days into starvation. One explanation for these embryos is that they represent recent fertilizations, occurring in animals that did not begin embryo production for up to five days. An alternate explanation, as suggested previously [11], is that these embryos are in a state of embryonic arrest or slowed development, with the embryos remaining in early embryogenesis for up to five days, and with the parent hermaphrodite ceasing additional ovulations.

To distinguish between these possibilities, we tracked the fate of individual animals containing one or two viable-looking, early-stage embryos on day 3 of starvation. To track single animals, we first starved parallel populations of wild-type and GFP-expressing animals from ‘Mid/early’ L4. On day 3 of starvation, we transferred a total of 261 GFP-expressing animals into separate, starved populations of wildtype. Animals chosen for transfer contained one or two viable-looking, early-stage embryos in utero and no late-stage embryos or hatched L1s. On day 4, we recovered the GFP-expressing animals and recorded the fates of their embryos.

**Figure 2:**
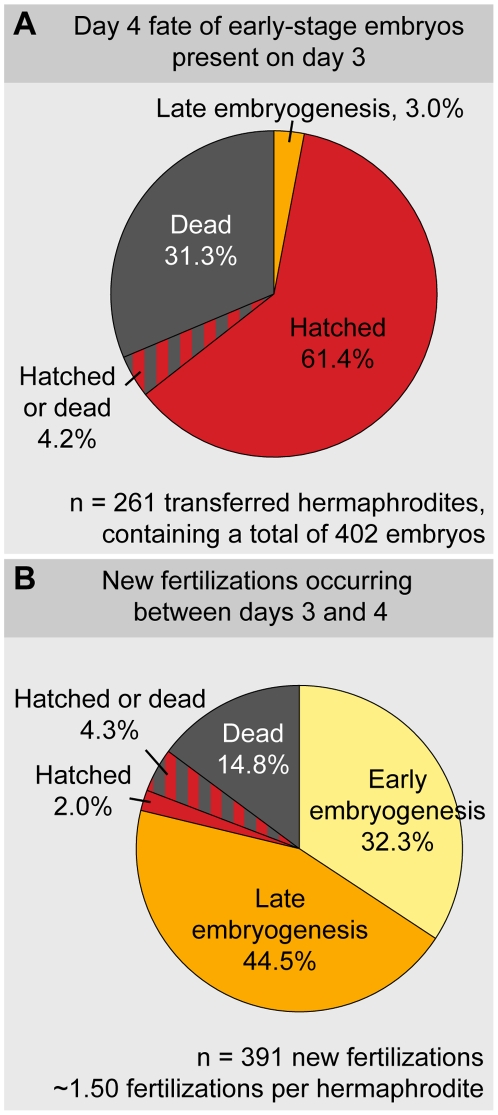
Embryos produced during starvation do not arrest. Animals containing one or two viable-looking, early-stage embryos were tracked in crowded populations during days 3 to 4 of starvation. (A) Fates on day 4 of starvation of embryos identified on day 3. (B) New fertilizations, shown according to the stage of each newly fertilized embryo on day 4. (A–B) ‘Hatched or dead’ refers to cases where one embryo present before transfer and one newly fertilized embryo (observed on day 4) had either hatched or died, but we were not able to not determine which embryo had adopted which fate. A more detailed classification of the developmental stages is shown in Figure S2.

The results of this experiment demonstrate that animals containing one or two viable-looking, early-stage embryos on day 3 of starvation do not remain in this state for even one full day (Figure 2). By day 4 of starvation, the majority of tracked embryos had either hatched or died: At least 61.4% (*n* = 402) of these embryos hatched, and at least 31.3% died; none remained in early embryogenesis (Figure 2A, Figure S2A). This lethality was not an effect of the transfer procedure because early-stage embryos dissected from similarly starved hermaphrodites died at a comparable rate: 37.6% (*n* = 753). Thus, early-stage embryos present on day 3 of starvation do not remain in early embryogenesis on day 4; instead, these embryos either hatch within a ∼24 h period or die. This timing is consistent with these embryos developing at the normal rate of ∼14 h between fertilization and hatching.

Moreover, the tracked hermaphrodites continued to ovulate: By day 4 of starvation, 88% (*n* = 261) of the tracked hermaphrodites had produced at least one newly fertilized embryo (Figure 2B). Embryos were produced at a rate of 1.50±0.78 embryos per hermaphrodite per ∼24 h period (Figure S2C), over 80-fold slower than the rate in a fed animal [12]. Like the embryos present on day 3, the newly fertilized embryos did not remain in early embryogenesis: At the time of recovery (on day 4), 46.5% (*n* = 391) of the newly fertilized embryos had already entered late embryogenesis or had hatched (Figure 2B, Figure S2B). Thus, animals containing one or two viable-looking, early-stage embryos on day 3 of starvation continue to ovulate, and their embryos continue to develop. We conclude that embryos produced during starvation do not enter a state of embryonic arrest or slowed development; instead, early-stage embryos visible during starvation represent recent fertilizations.

### Germline shrinkage occurs in all starved, oogenic adults

**Figure 3:**
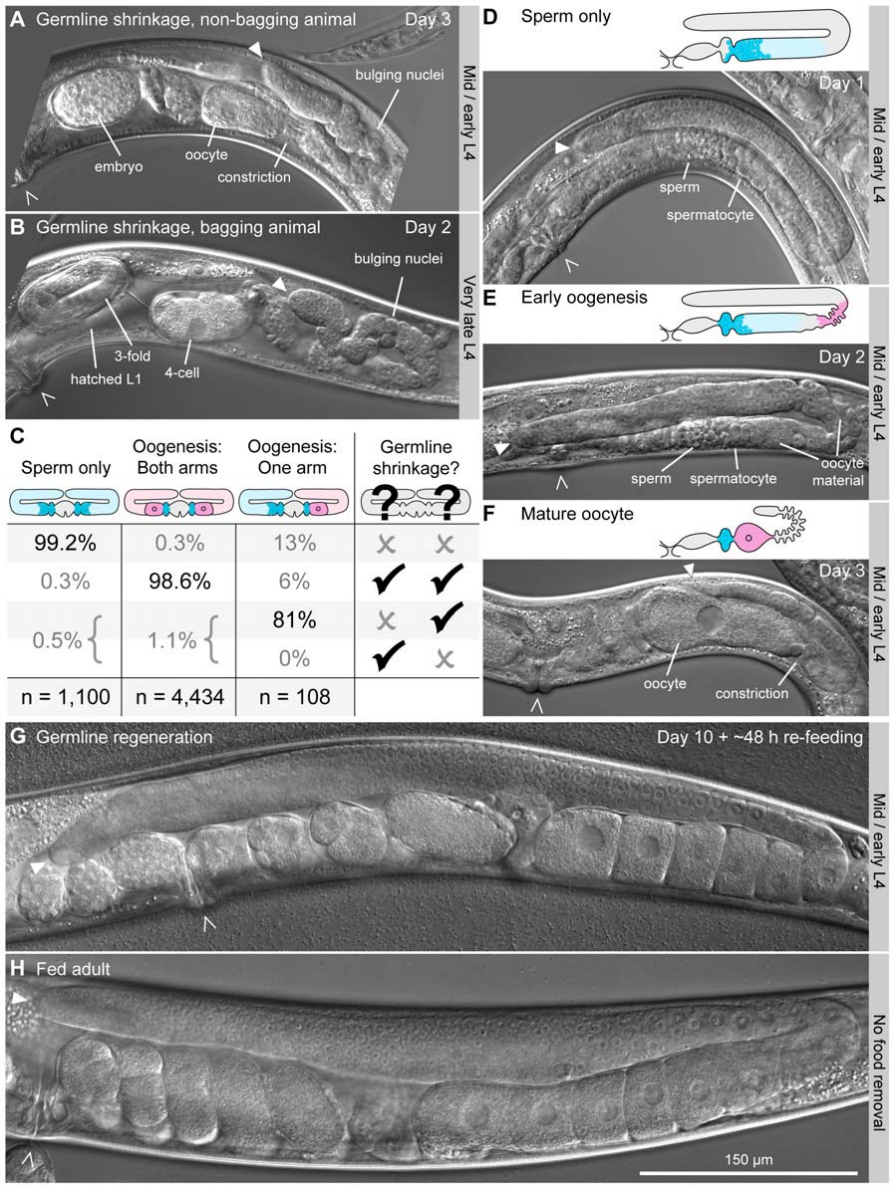
All hermaphrodites starved from L4 shrink their germlines once oogenesis begins. (A) Germline shrinkage in non-bagging animal. The embryo in utero looks viable. (B) Germline shrinkage in a bagging animal. The uterus contains a 4-cell embryo and 3-fold embryo. A hatched L1 is out of focus but visible. (C) Correlation between germline shrinkage and the onset of oogenesis. Starved adults across all ages at food removal (Figure 1A) were classified according to (i) the onset of oogenesis in each germline arm and (ii) the presence or absence of germline shrinkage in each arm. Presence or absence of shrinkage is indicated by check-marks and X-marks, respectively. Percentages sum within columns, with the total number of animals scored indicated at the bottom (*n*). (D–F) Germline shrinkage begins in the same location as oogenesis. In schematic diagrams, pink indicates oocyte material, light blue indicates spermatocytes, and dark blue indicates sperm. Furrowed outline of the germline indicates shrinkage. (D) Adult germline that has not yet begun oogenesis. No shrinkage is visible. (E) Adult germline in early oogenesis. Oocyte material is visible in the bend of the germline and just proximal to the bend. Shrinkage is present in these locations as well, visible as a furrowing of the exterior surface of the germline. (F) Adult germline containing a mature-sized oocyte and no embryos. This germline is shorter than the germline in panel (E) (i.e. more shrunken). A constriction is visible behind the single oocyte. (G) Regenerated germline in an animal starved for 10 days then re-fed for ∼48 h. (H) Germline in a fed adult that had never been starved, shown for comparison. This image was acquired on day 2 of adulthood. (A–B, D–G) All images are the shown at the same magnification; starved animals are shorter and thinner than fed adults. For each animal, the vulva and distal tip of the germline are marked by a caret and an arrowhead, respectively. For starved animals, the age at food removal is indicated to the right of each panel; the day of starvation on which the image was acquired is indicated on each image.

Next, we examined germline morphology in starved animals. Previously it was reported that during starvation from the L4 stage, non-bagging adults shrink their germlines [11]. We have confirmed this finding: Starved adults were smaller than fed adults (Figure 3A vs. 3H), and their germlines were shorter relative to the length of the animal (Figure 3A). In addition, the cytoplasmic core of the germline was dramatically reduced in volume (Figure 3A, Figure S3A–J), and germ cells “bulged” out from the rest of the germline, appearing as if cytoplasm surrounding these cells had been reduced (Figure 3A, Figure S3B). The distal germline seemed protected from shrinkage and almost never exhibited bulging (Figure 3A, Figure S3A–B).

We have extended this finding to learn that germline shrinkage is not unique to animals surviving starvation: All adults containing embryos or L1s possessed shrunken germlines (*n* = 3,468). Shrinkage occurred regardless of the age at food removal, and regardless of the bagging fate (Figure 3B, Figure S3A–H). Germline shrinkage was also observed in animals starved from young adult (Figure S3J), although shrinkage in these animals progressed more slowly and could not be tracked after day 1 of starvation because these animals bagged so quickly.

Germline shrinkage was strongly correlated with oogenesis. Non-oogenic adults rarely showed germline shrinkage (Figure 3C–D); oogenic adults almost always did (Figure 3C,E–F); and animals that had begun oogenesis in one germline arm but not the other typically showed shrinkage in the oogenic germline arm only (Figure 3C). (We define ‘shrinkage’ as furrowing of the exterior surface of the germline. See Materials and Methods for more detail.) Moreover, the initial location of germline shrinkage coincided with the initial location of oocyte growth: Shrinkage was first observed at the bend of the germline, the same location where oogenic material first accumulates (Figure 3E). As oogenesis proceeded, germline shrinkage became progressively more severe (Figure 3F). Shrinkage seemed to progress distally, from the bend of the germline, because shrinkage was sometimes observed in the proximal germline only (Figure S3E), but never the reverse scenario. We conclude that although all hermaphrodites starved from L4 shrink their germlines as adults, shrinkage does not begin until oogenesis.

### Oocyte growth during starvation

In contrast to fed animals, starved animals almost always formed only a single oocyte at a time per gonad arm: Of 8,976 oogenic arms examined during starvation, 99.94% contained no more than a single oocyte; fed animals, in contrast, typically form six to ten oocytes per gonad arm (Figure 3H). Moreover, in starved animals, the single oocyte was typically separated from the remainder of the germline by a tight constriction, with oocyte material confined to the proximal side (Figure 3F, Figure S3C). Occasionally, oocyte material occurred on both sides of the constriction (Figure S3D), and in such cases, we could observe transport of material across the constriction via cytoplasmic flow. Additionally, we witnessed two ovulations in starved animals, and following each ovulation, no oogenic material remained distal to the spermatheca. Likewise, no oogenic material was observed distal to the spermatheca in germlines containing 1-cell embryos (*n* = 194) (Figure S3F). These observations imply that during starvation, growth of each oocyte begins only after ovulation of the previous oocyte is complete.

Consistent with oocytes forming sequentially, rather than simultaneously, ovulations in starved animals were spaced far apart in time: After day 1 of starvation, among germlines in which the most recently fertilized embryo was at the 6-cell stage or earlier, 99.5% (*n* = 208) of adjacently fertilized embryos were in late embryogenesis, had hatched, or were dead (Figure S3F). Given that embryos enter late embryogenesis ∼8 h post-fertilization, the infrequency of adjacent early-stage embryos indicates that ovulations in starved animals usually occurred at least 8 h apart. This timing is much less frequent than in a fed animal, where ovulations occur at a rate of one ovulation every ∼23 min per gonad arm [12].

Consistent with ovulations occurring at least 8 h apart, the complete growth of each oocyte often required 8 h or more: Among germlines in which the most recently fertilized embryo was in late embryogenesis or had hatched, 74.6% (*n* = 2,181) of germlines contained oocyte material totaling less than half the volume of a mature oocyte (Figure S3H). Given that animals form only a single oocyte at a time per germline arm, this result indicates that even after 8 h of growth, these oocytes were still in the early stages of development. Thus, the process of making a fully mature oocyte usually required at least 8 h. We conclude that during starvation, animals ovulate infrequently, and the rate of ovulation is limited by the rate of oocyte growth.

### Germlines regenerate upon re-feeding

**Figure 4:**
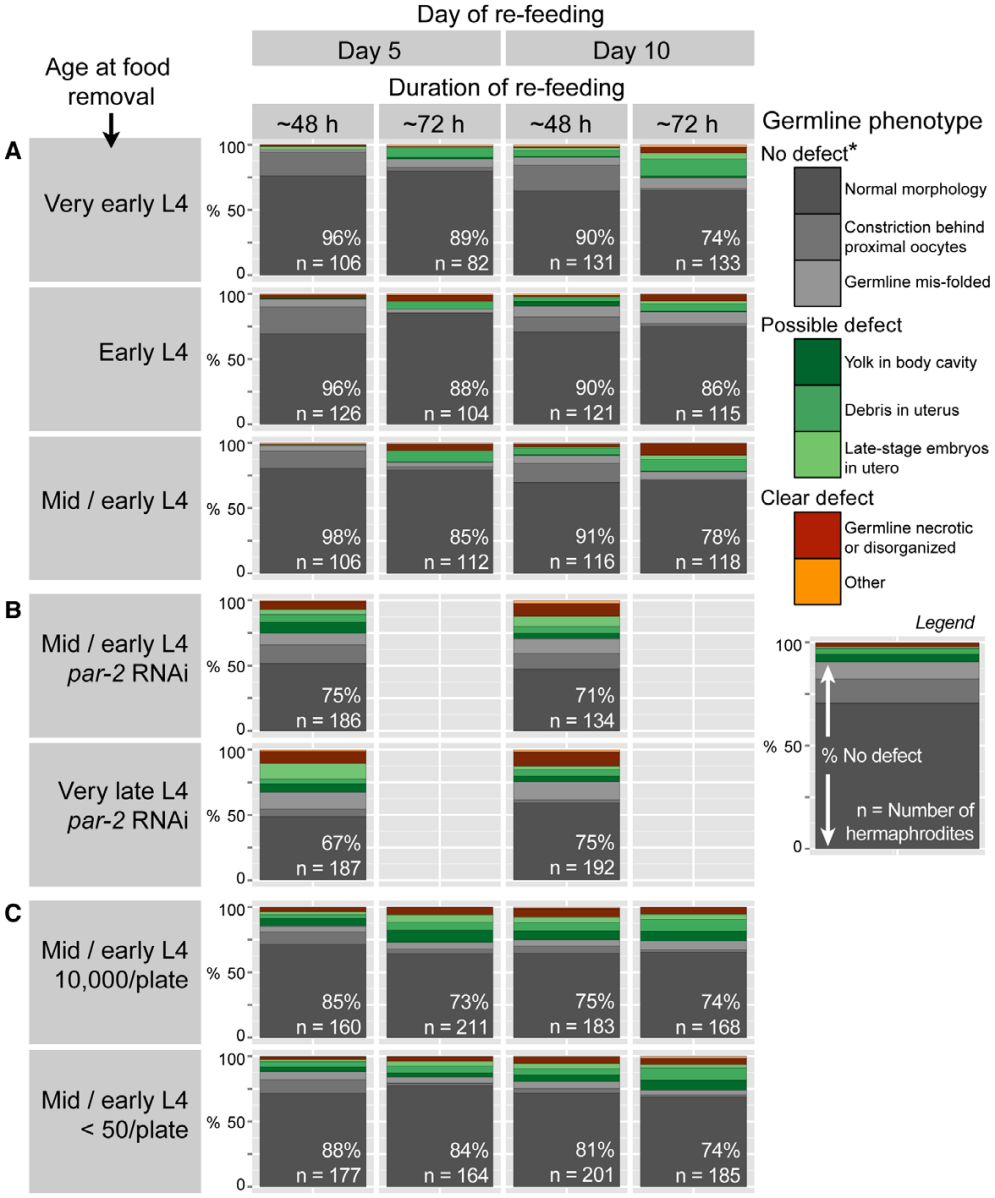
Most starved hermaphrodites are capable of germline regeneration upon re-feeding. (A) Germline regeneration in animals starved from ‘Very early’ to ‘Mid/early’ L4. (B) Germline regeneration in animals grown on *par-2* RNAi prior to starvation and starved from ‘Mid/early’ or ‘Very late’ L4. In (A–B), populations were starved at a density of ∼10,000–14,000 animals per 10 cm plate. (C) Germline regeneration in animals starved from ‘Mid/early’ L4 at densities of ∼10,000 animals per 10 cm plate and less than 50 animals per plate. High- and low-density plates were prepared in parallel. In all panels, stacked bar graphs show the fraction of germlines in each phenotypic category. Germline arms showing multiple abnormalities are plotted according to the more severe abnormality. The number of hermaphrodites examined is indicated on each graph. For each hermaphrodite, both germline arms were scored. Percentages in white indicate the fraction of germline arms classified as ‘No defect.’ *, Stacked oocytes or unfertilized oocyte material in utero were not classified as defects. See Materials and Methods for a detailed description of germline phenotypes.

We next investigated how the shrunken germlines respond to re-feeding. Previously, it was reported that when surviving animals are re-fed, their shrunken germlines regenerate [11]. We have confirmed this finding: Upon re-feeding of animals starved from ‘Very early’ to ‘Mid/early’ L4, after 5 or 10 days in starvation, 74%–98% (*n* = 164–266) of germlines regenerated with essentially normal morphology (Figure 3G, Figure 4A).

The regenerated germlines resembled the germlines of a fed young adult (Figure 3G vs. 3H). Three exceptions to this rule are the following. First, the constriction present during starvation sometimes remained after re-feeding, albeit in a more relaxed state (Figure S4A–B). Constrictions persisted more often after ∼48 h of re-feeding than ∼72 h (Figure 4), suggesting that relaxation of the constriction occurred over several days. Second, the regenerated germlines sometimes contained stacked oocytes or unfertilized oocyte material in utero (Figure S4A), suggesting that sperm had not survived starvation well. This observation is consistent with [11], who reported that after re-feeding, production of self-progeny but not cross-progeny was reduced. Third, the regenerated germlines were occasionally mis-folded (Figure S4C), perhaps because the contortions induced by germline shrinkage did not correct themselves during regeneration.

Given that germline shrinkage was not unique to animals surviving starvation, we next tested whether animals that normally bag would instead survive and regenerate their germlines if bagging were prevented. To test this possibility, we starved animals from ‘Very late’ L4, which bag at a rate of 95%–97% (Figure 1A); however, prior to starvation, we grew the animals on bacteria expressing *par-2* (*F58B6.3*) RNAi, which kills embryos in utero [13], [14]. When grown on *par-2* RNAi, animals starved from ‘Very late’ L4 were able to survive starvation, and these animals regenerated their germlines at rates similar to control animals starved from ‘Mid/early’ L4 (Figure 4B, Figure S3K). Thus, the capacity for germline regeneration is shared by virtually all animals.

Finally, we noticed that starved animals could resume embryo production without regenerating their germlines. On three occasions, populations starved from ‘Very early’ to ‘Mid/early’ L4 became contaminated with an unknown species of bacteria on or near day 10 of starvation. This contaminant did not form a lawn on the plate, but instead formed small patches of sparsely distributed cells, visible only under high magnification; most animals were therefore not in contact with this food source at any given time. Animals on these plates still appeared starved in that egg-laying was inhibited, and animals remained pale and thin. In addition, germlines did not regenerate (*n* = 136 germlines). However, animals resumed oocyte and embryo production: In the contaminated populations, 24% (*n* = 68) of oogenic animals contained mature-sized oocytes, and 54% contained viable-looking embryos or hatched L1s (0.87±0.99 non-dead progeny per animal); multiple oocytes per germline arm were never observed (n = 136 germlines), and consistent with egg-laying being inhibited, 49% (*n* = 59) of the non-dead progeny in utero were late-stage embryos or hatched L1s. By contrast, in parallel populations lacking the contaminant, the comparable rates of mature-sized oocytes and non-dead progeny were 0.0% (*n* = 99) and 4.0%, respectively. One possible explanation for these findings is that when starved animals encounter a small amount of food, they resume embryo production but do not regenerate their germlines. This explanation is consistent with the findings of [11], who reported that low-level plate contamination caused starved animals to bag without regenerating their germlines. Further experiments are needed to determine the quantity and quality of food required for oocyte production, germline regeneration, and the resumption of embryo production.

### Effect of crowding

We next tested the effect of crowding. We removed food at ‘Mid/early’ L4 and plated animals at two densities in parallel: Approximately 10,000 animals per 10 cm plate and less than 50 animals per plate. By three parameters measured, animals at high and low density responded to starvation equivalently: Animals at low density delayed embryo production (Figure S5); animals at low density showed germline shrinkage (*n* = 620 oogenic germlines) (Figure S3I); and animals at high and low density regenerated their germlines at similar rates (Figure 4C). We conclude that crowding does not play a major role in the starvation response of L4 hermaphrodites.

## Discussion

This work characterizes the starvation response of ‘Very early’ to ‘Very late’ L4 hermaphrodites. Our results support four major conclusions. First, starvation delays the onset of reproduction and reduces embryo viability. This reduction in embryo viability allows some animals to avoid the bagging fate and survive long term. Second, all animals respond to starvation equivalently with respect to germline shrinkage and germline regeneration: All oogenic germlines shrink during starvation, and when bagging is prevented ectopically, virtually all germlines regenerate upon re-feeding. Third, germline shrinkage strongly correlates with oogenesis. Fourth, the starvation response does not require crowding. These conclusions have major implications for our understanding of how animals reproduce when nutrients are limited. In addition, these conclusions confirm some but not all phenomena described in a similar study [11]; most importantly, our conclusions do not support the existence of a specialized program of reproductive dormancy.

### Re-thinking Adult Reproductive Diapause

In a previous study examining starvation from the L4 stage, Angelo and Van Gilst [11] reported a pioneering set of observations revealing the existence of germline shrinkage and germline regeneration. Their study found that: (i) animals starved from L4 sometimes survive as adults; (ii) populations starved from L4 contain non-bagging adults with one or two viable-looking, early-stage embryos up to five days into starvation; and (iii) non-bagging adults shrink their germlines during starvation and regenerate their germlines upon re-feeding [11]. Our results corroborate these key findings.

**Figure 5:**
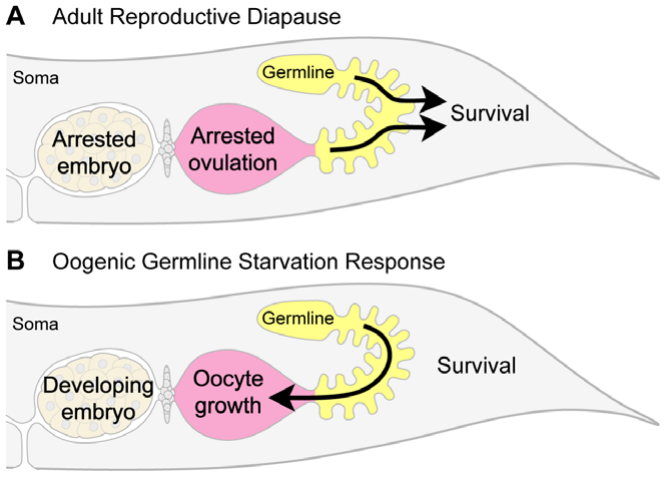
Adult Reproductive Diapause and the Oogenic Germline Starvation Response propose alternate functions of germline shrinkage. (A) The model of Adult Reproductive Diapause [11] proposes that the primary role of germline shrinkage is to provide fuel for adult survival (arrows from shrunken germline to adult survival). According to this model, animals enter state of reproductive dormancy when starved at high density; in this state, the animal produces at most one embryo per gonad arm; ovulations then cease, and embryos remain in early embryogenesis for up to five days, before eventually dying. (B) The model of the Oogenic Germline Starvation Response (this work) proposes that the primary role of germline shrinkage is to provide material for oocyte production (arrow from shrunken germline into growing oocyte). According to this model, all animals respond to starvation equivalently, with survival of the parent hermaphrodite dependent on its failure to make viable embryos during starvation. Embryos continue to develop, and hermaphrodites continue to ovulate, albeit at a reduced rate; by day 10 of starvation, ovulations largely cease, and embryos in surviving animals are all dead. Population density plays no major role in the starvation response. Remodeling of somatic tissue likely contributes to both adult survival and oogenesis but is not included in either diagram.

Angelo and Van Gilst [11] also proposed that hermaphrodites survive starvation by entering a state of reproductive dormancy, akin to the dauer larval stage [15] or L1 arrest [16], [17]. They termed this state Adult Reproductive Diapause (Figure 5A) and describe it as having four distinguishing features: (i) germline shrinkage during starvation; (ii) capacity for germline regeneration upon re-feeding; (iii) in utero embryonic arrest or slowed development; and (iv) a requirement for crowding [11]. According to their model, this diapause state represents a regulatory program distinct from the bagging fate, such that animals starved from L4 make a life-history decision between diapause and bagging [11].

Our results confirm the existence of germline shrinkage and germline regeneration. Yet three observations lead us to question the existence of a distinct diapause state. First, germline shrinkage occurs in both bagging and non-bagging animals; moreover, if bagging is prevented by *par-2* RNAi, virtually all germlines regenerate. Thus, germline shrinkage upon starvation and germline regeneration upon re-feeding are not limited to a small subset of animals entering a specialized state of dormancy.

Second, embryos produced during starvation do not arrest. Previous evidence for embryonic arrest came from the observation that starved populations contain non-bagging adults with one or two viable-looking, early-stage embryos on up to five days into starvation [11]. Our results show that these embryos are not arrested but instead represent recent fertilizations, occurring in animals in which embryo production was delayed.

Third, crowding does not affect the starvation response: We find that animals starved at high or low density (10,000 vs. 50 per plate) respond to starvation equivalently. Previous evidence for crowding relied on the observation that starved animals transferred individually to fresh plates usually bagged [11]; this observation was interpreted as evidence that the proposed embryonic arrest required crowding [11]. Given the absence of embryonic arrest, however, we suggest that the animals transferred individually to fresh plates might have also bagged on a crowded plate, if they had been tracked there. We conclude that crowding does not play a major role in the starvation response of L4 hermaphrodites.

In summary, three of the proposed features of Adult Reproductive Diapause [11] either do not exist (embryonic arrest and a requirement for crowding) or are shared by animals adopting the bagging fate (germline shrinkage); the fourth feature (germline regeneration) is also shared by animals that normally bag, when bagging is prevented ectopically. Thus, we conclude that all L4 hermaphrodites respond to starvation equivalently: The oogenic germline shrinks; the animal produces as many embryos as possible, given nutrients available; and the viability of those embryos determines adult survival. All animals retain the capacity for germline regeneration, but only animals surviving starvation are able to realize it. The increased survival rates for animals starved from early versus late L4 simply reflect the decreased ability of animals starved from early L4 to make viable progeny during starvation.

How should we refer to the starvation response of L4 hermaphrodites? One option is to use the term ‘Adult Reproductive Diapause;’ this option poses two problems. First, this term was defined by features that have not been supported experimentally. Second, this term cannot be applied to all animals, because bagging animals do not enter a ‘diapause’ – they die. We therefore propose an alternate term, the ‘Oogenic Germline Starvation Response,’ to describe the germline changes accompanying starvation. We define this response by (i) germline shrinkage that coincides with oogenesis, and (ii) capacity for germline regeneration upon re-feeding. Defined in this way, all hermaphrodites starved from L4 execute the Oogenic Germline Starvation Response. In addition, this response is associated with several other reproductive changes: Increased embryo lethality; slower oogenesis; reduced ovulation rate; formation of a single oocyte per germline arm; and the appearance of a tight constriction behind the single oocyte.

Although our results do not support Adult Reproductive Diapause as defined by [11], starvation clearly induces a broad spectrum of changes involving both the germline and soma. The Oogenic Germline Starvation Response is only part of this response; animals surviving a lengthy period of starvation undoubtedly experience a vast array of additional changes, likely affecting both their metabolic program and cellular physiology. Referring to these changes as ‘Adult Reproductive Diapause’ may prove useful, provided that this term is redefined by a different set of features – features that accurately distinguish long-term survivors from animals that bag.

### Function of the oogenic germline starvation response

The correlation between germline shrinkage and oogenesis suggests that during starvation, germline shrinkage facilitates oocyte production. One simple model for shrinkage is that during starvation, the animal scavenges material from its germline to produce oocytes (Figure 5B). This model is consistent with several observations: Germline shrinkage does not occur until the onset of oogenesis; shrinkage begins in the same location where oogenic material first accumulates; shrinkage becomes more severe as starvation proceeds; and shrinkage seems to progress in the proximal-to-distal direction. In addition, during starvation, we directly observed transport of material from the germline into the growing oocyte via cytoplasmic flow.

We hypothesize that somatic tissues might also contribute material to the oocyte. Adults starved from L4 are shorter and thinner than fed adults, but we do not know whether the soma actually shrinks during starvation or simply fails to grow from its L4 size. The prolonged time-to-completion of each oocyte may reflect the additional time required to mobilize nutrient stores (from the germline and soma) and transform them into oocytes. Likewise, the eventual cessation of oocyte production by day 10 of starvation may reflect the eventual depletion of nutrient stores. Finally, the decreased ability of hermaphrodites starved from early L4 to produce viable progeny during starvation likely reflects these animals being smaller at the onset of starvation and therefore having less material available for oogenesis.

Our model for germline shrinkage differs from the model proposed by [11], who suggested that the primary function of germline shrinkage was to fuel adult survival during starvation (Figure 5A). We cannot exclude adult survival as a contributor to germline shrinkage, however, the strong correlation between oogenesis and germline shrinkage suggests that the primary role of shrinkage is oocyte production. In addition, the model of germline shrinkage fueling adult survival [11] is inconsistent with the observation that animals seem to prioritize reproduction over survival: Despite the risk of bagging, the vast majority of animals starved from L4 try to reproduce. The ecological relevance of bagging remains unclear, although bagging might provide a nutritional advantage to progeny [9], [10].

### Fundamental questions now tractable: Future directions

This work raises several fundamental questions about how reproduction is regulated upon starvation. One key feature of the Oogenic Germline Starvation Response is germline shrinkage, which we suggest depends on two cellular processes: Depletion of germ cell number and depletion of germline cytoplasm. Neither process is understood. Depletion of germ cell number likely occurs through cessation of cell divisions in the distal germline and increased apoptosis in the pachytene region; such effects occur when adult hermaphrodites are starved for only 6 h [18], and complete germline shrinkage in animals starved from L4 requires the apoptosis-promoting caspase *ced-3* [11]. Importantly, reduction in germ cell number upon starvation occurs without loss of the germline stem cell pool [11, this work], indicating that the Oogenic Germline Starvation Response uncouples stem cell maintenance from germline proliferation. One explanation for this uncoupling is that down-regulation of proliferation might depend on the program of oogenesis.

Depletion of germline cytoplasm upon starvation might depend on several factors: Transport of cytoplasm into the single developing oocyte; decreased yolk production in the intestine, leading to decreased yolk uptake by the germline; and export of material from germline cytoplasm into the soma. The first mechanism – loss of germline cytoplasm into the oocyte – almost certainly contributes to germline shrinkage because oocyte production continues during starvation, with germline cytoplasm flowing into the developing oocyte. Moreover, this mechanism may account for the absence of shrinkage in the distal germline, where GLP-1/Notch signaling inhibits cytoplasmic streaming [19]. However, understanding the balance between this mechanism and others will require additional experiments tracking the movement of material within the germline and between germline and soma.

Other questions raised by our study include: How are developing oocytes regulated to grow to the appropriate size before maturation? How does a constriction form behind the single oocyte, and what is the function of this structure? We speculate that starved gonads monitor oocyte volume, although the mechanism remains unclear. We also speculate that the constriction may act to prevent backflow of material from the oocyte into the distal germline; this constriction might be formed by the myo-epithelial sheath cells, which encase the germline. Finally, how is germline regeneration regulated upon re-feeding? Does regeneration rely on the same regulators that control germline self-renewal? Fortunately, the Oogenic Germline Starvation Response does not require crowding, and its features occur in all starved hermaphrodites, not just a small subset as suggested by the model of Adult Reproductive Diapause [11]. Thus, these fundamental questions in reproductive biology can now be analyzed by the full toolkit of modern genetics.

## Materials and Methods

### *C. elegans* strains and culture methods

The following strains were used: N2 and CB5584 *mIs12*[*myo-2::GFP*]. Unless otherwise noted, animals were maintained at 20±0.5°C on nematode growth media (NGM) plates seeded with *E. coli* OP50 as a food source. Our nematode growth media contained, per 1 L of media: 3 g NaCl, 2.5 g peptone, 20 g agar (Sigma-Aldrich #A7002), 25 ml 1 M Potassium Phosphate Buffer (pH = 6.0) [20], 1 ml 1 M CaCl_2_, 1 ml 1 M MgSO_4_, 1 ml 5 mg/ml cholesterol (in 95% ethanol), and 1 ml 2 mg/ml uracil.

### Live imaging

Imaging was performed by mounting animals in M9 [20] on 4% agar pads. When anesthetization was required, animals were mounted in 30 mM sodium azide in M9. All images were acquired at 63× magnification on a Zeiss AXIO Imager.D1 microscope equipped with a Hamamatsu C4742-95 camera. When multiple images were needed to cover the subject of interest, images were aligned using the Photomerge function in Adobe Photoshop CS4.

### Starvation protocol

Synchronous L1s were obtained by hypochlorite-treating gravid hermaphrodites in a 12∶2∶1 solution of M9∶Clorox bleach∶5M NaOH for 7–8 minutes, with vortexing every minute. The resulting embryos were washed 3 times in M9 and then incubated for ∼14 h at 22°C, with 175 rpm shaking, in 500 ml flasks containing ∼150 ml of S-media [20] per flask. The concentration of embryos in these flasks did not exceed 500 embryos/ml. The hatched L1s were then collected, washed 2 times with M9, and plated on 10 cm plates (seeded with OP50), at a density of ∼1,200 L1s per plate. To ensure uniform feeding of L1s, OP50 on these plates was spread over >90% of the plate. Plates were incubated at 20±0.5°C until the onset of food removal.

After ∼32 h of incubation, animals were monitored every 30 minutes to determine when populations had reached ‘Very early’ L4. Monitoring was performed by (i) scoring animals as ‘dark’ or ‘pale’ to determine whether they had undergone the L3 to L4 molt, and (ii) examining a subset of animals at 63× magnification to determine the extent of gonad growth. Populations were defined as ‘Very early’ L4 when more than half the population was ‘pale,’ and gonad growth in the majority of animals matched that shown in Figure 1A. ‘Early’ to ‘Very late’ L4 (Figure 1A) were defined as occurring at 2 h intervals after ‘Very early’ L4. By this definition, populations at ‘Very early’ L4 included some L3 animals, but populations at ‘Very late’ L4 did not contain any adults. ‘Young adult’ was defined as occurring 4 h after ‘Very late’ L4; at this timepoint, the vast majority of animals had molted into adulthood.

In our hands, populations reached ‘Very early’ L4 at ∼33.5 h after release from L1 arrest. (Thus, populations reached ‘Very late’ L4 at ∼43.5 h and ‘Young adult’ at ∼47.5 h.) However, the timing of development was variable and depended upon several factors: The exact length of the L1 arrest; the exact temperature of the incubator; the temperature of the plates onto which L1s were plated (pre-equilibrated to 20°C versus at room temperature); and how quickly the plates cooled down to 20°C, if they had not been pre-equilibrated. In addition, populations were not perfectly synchronous at the onset of starvation: From one timepoint to the next, the average stage of the population was shifted by 2 h, but stages of individual animals overlapped. We suspect that this variability accounts for much of the variation in the timing of the molt into adulthood, the onset of oogenesis, and the onset of embryo production within each ‘Age at food removal’ (Figure 1D).

To initiate starvation, ∼5 ml M9 was added to each 10 cm plate, and animals were dislodged by shaking the plates at ∼20 rpm for 5 minutes. The resulting solution was then transferred into 15 ml conical tubes (∼3–7 plates per tube) and spun at ∼450 g in a swinging bucket rotor for 1 min. The supernatant was aspirated, and animals were washed 6 additional times. For each wash, ∼10 ml M9 was used per tube, and animals were spun at ∼450 g for 1 min. Typically, after the third wash, all turbidity was removed.

Animals were then plated on 10 cm starvation plates, at a density of ∼10,000–14,000 animals per plate. Our starvation plates contained, per 1 L of media: 3 g NaCl, 25 g agar (Sigma-Aldrich #A7002), 25 ml 1 M Potassium Phosphate Buffer (pH = 6.0), 1 ml 1 M CaCl_2_, 1 ml 1 M MgSO_4_, and 1.6 ml 5 mg/ml cholesterol (in 95% ethanol). Peptone was excluded from starvation plates to reduce the residual growth of any remaining bacteria. Extra agar and cholesterol were added for consistency with [11].

### Estimating survival rates and progeny production

For each ‘Age at food removal’ (Figure 1A), nine populations of ∼13,700 animals were starved in parallel. Each starved population was plated onto a single 10 cm plate. On days 3, 5, and 10 of starvation, animals from three plates were washed into separate 15 ml conical tubes with M9+0.1% Triton X-100. Volumes were brought up to 12 ml. From each tube, 10 aliquots of 0.2 ml were removed, and the number of surviving animals was counted in each aliquot. These counts were then averaged and back-calculated to obtain single estimates of the number of surviving animals per plate.

To estimate the total number of progeny produced by day 10, an additional 6 aliquots of 0.2 ml were removed from tubes on day 10, and the number of L1s, L2s, and dauers was counted in each aliquot. Again, these counts were averaged and back-calculated to obtain single estimates per plate. These estimates were then divided by 13,700 to normalize by the total number of starved animals.

### Sampling animals from starved populations

Surviving hermaphrodites were collected from each starved population in mass and examined at 63× magnification; bagged carcasses and occasional spontaneous males were excluded. Hermaphrodites were classified as L4 or adult according to vulval morphology and whether cuticle covered the vulva opening. Animals wearing a half-shed cuticle were classified as adults. Adults were further classified as ‘Sperm only’ or ‘Oogenic’ according to the presence or absence of oocyte material in each germline arm. We define oocyte material as granular cytoplasm characteristic of oocytes. At the onset of oogenesis, oocyte material was typically first observed at the bend of the germline or just proximal to the bend.

In oogenic animals, we recorded the number of oocytes in each germline arm and the volume of oocyte material in each oocyte. Volumes were classified as (i) less than half the volume of a mature oocyte; (ii) equal to the volume of a mature oocyte; or (iii) somewhere in between. Estimates were made by eye and are therefore crude. In all cases, we erred on the side of overestimating volumes.

In animals containing embryos or hatched L1s, we recorded the total number of embryos or hatched L1s present. We also classified each embryo as (i) 6-cell stage or earlier; (ii) older than 6-cell but younger than comma-stage; (iii) comma-stage to 2-fold; (iv) >2-fold; or (v) dead. Our definition of ‘dead’ was very conservative: We feel confident that we never mistook a normal-looking embryo for a dead one, but we probably did mis-classify some dead embryos as ‘viable-looking.’

For two reasons, the fraction of dead embryos contained in sampled animals does not equal the true rate of embryo lethality. First, embryos may appear ‘viable-looking’ in early embryogenesis but still exhibit defects later in development. (In fact, defects in embryos produced during starvation seemed to be highly variable.) This bias will cause the fraction of dead embryos in sampled animals to underestimate of the true rate of embryo lethality. Second, once animals begin to die via bagging, samples are necessarily biased towards animals that have produced fewer viable embryos. This bias will cause the fraction of dead embryos in sampled animals to overestimate of the true rate of embryo lethality. The relative strength of these biases will change over time because as more and more animals bag, samples will become more and more biased towards animals that have produced fewer viable embryos.

Finally, on each day of starvation, we recorded the presence or absence of L1s outside parent hermaphrodites. Because starvation inhibits egg-laying, and starved populations never contained laid embryos, the presence of these L1s can be used as an indicator that some adults have already died via bagging. Also, the appearance of L1s always coincided with the appearance of bagged carcasses.

### Scoring germline shrinkage

Animals were classified as showing germline shrinkage if any portion of the germline exhibited furrowing of the exterior surface of the germline, as shown in Figure 3A–B and 3E–F and Figure S3A–J. Furrowing was typically first observed at the bend of the germline or just proximal to the bend. Furrowing generally preceded regression of the distal germline away from the vulva. Reduction in volume of the cytoplasmic core occurred coincident with furrowing; however, even at the onset of furrowing, germlines in starved animals were narrower than in fed adults, reflecting the smaller body size of starved animals. In addition, circumferences of the germline at the onset of furrowing depended upon the age at food removal: Circumferences were smaller in animals starved from early L4 versus late L4, presumably reflecting smaller body sizes and smaller germline volumes at the onset of starvation. In animals starved from young adult, all aspects of shrinkage were less severe: Furrowing was shallower; cytoplasmic cores were wider; and distal germlines regressed less. We suspect that in animals starved from young adult, germline shrinkage would have eventually become more severe, if we had been able to follow animals after day 1 of starvation; however, nearly all animals starved from young adult bagged by day 2.

Spontaneous males were occasionally observed and never exhibited germline shrinkage (*n*>15). Likewise, shrinkage was never observed in L4s (*n* = 973).

### Definition of ‘early-stage’ and ‘late-stage’ embryos

We define ‘early-stage’ embryos as pre-comma stage embryos and ‘late-stage’ embryos as comma or post-comma. Thus, ‘early-stage’ and ‘late-stage’ correspond to the first ∼8 h and last ∼6 h of embryogenesis, respectively. We have chosen the comma stage to delineate ‘early’ versus ‘late’ because the comma stage is easy to recognize, and the comma stage corresponds to the stage when nearly all embryonic cell divisions are complete.

### Tracking single animals on a crowded plate

N2 and CB5584 *mIs12*[*myo-2::GFP*] animals were starved from ‘Mid/early’ L4 in parallel. After food removal, CB5584 animals were plated on starvation plates at a density of ∼10,000 animals per 10 cm plate. N2 animals were plated on 6 cm starvation plates at a density of ∼3,500 per plate (roughly equivalent to ∼10,000 per 10 cm plate). This modification was performed to reduce the number of N2 animals needed from ∼3 million to ∼1 million.

On day 3 of starvation, CB5584 animals were mounted individually on 4% agar pads in M9 and examined at 63× magnification. Animals containing one or two viable-looking, early-stage embryos and no late-stage embryos or hatched L1s were recovered from slides and transferred by mouth pipette into separate populations of N2. (These plates then contained ∼3,500 starved N2 animals and a single starved CB5584 animal.) Embryos within the transferred animals were counted at the time of transfer and classified according to the stages shown in Figure S2A. On day 4 of starvation (∼24 h after transfer), the CB5584 animals were recovered from N2 populations and examined at 63× magnification. The fates of all embryos (including new fertilizations) were again classified according to the stages shown in Figure S2A.

For most animals, we were able to determine a one-to-one mapping between the embryos present at transfer and embryos/L1s present upon recovery. In 17 cases, we were able to determine that one embryo present before transfer and one newly fertilized embryo had either hatched or died, but we were unable to determine which embryo had adopted each fate. These fates were therefore classified as ‘hatched or dead.’

In addition, we note that two types of mis-mappings may have occurred, both of which would have led us to underestimate the fraction of embryos present on day 3 that either hatched or died. First, dead embryos sometimes ruptured, and their remains were difficult to recognize. Thus, if an embryo present on day 3 died and ruptured by day 4, and if a new fertilization occurred in the same gonad arm, we may have mistaken the newly fertilized embryo for the embryo present in day 3. Second, hatched L1s occasionally exited the body of the parent hermaphrodite by day 4. These tiny L1s were extremely difficult to locate amongst the crowded population, and it is likely that we sometimes failed to find them. If this mistake did occur, and if a new fertilization occurred in the same gonad arm, we may have mistaken the newly fertilized embryo for the embryo present in day 3. We suspect that these mis-mappings may account for some (or all) of the 12 ‘late embryogenesis’ fates observed in Figure 2A.

Finally, we note that the rate of lethality among embryos present on day 3 is necessarily an underestimate of the true rate because our starting sample is biased against embryos that show abnormalities very early in development.

### Scoring lethality of dissected embryos

Embryos were dissected in mass on day 3 of starvation from animals starved at ‘Mid/early’ L4. Dissections were performed in M9, and after dissection, early-stage embryos were transferred by mouth pipette to 4% agar pads and covered with a cover-slip. Slides were then sealed with Vaseline. Immediately after mounting, embryos were examined at 63× magnification. Embryos that appeared abnormal, damaged, or dead were excluded from analysis. Slides were then incubated at 20±0.5°C. After ∼24 h, embryos were scored as hatched or dead. As a control, embryos were also dissected from animals starved at ‘Very late’ L4 on days 1 and 2 of starvation. The rates of embryo lethality for these controls were 4.8% (*n* = 165) for day 1 and 7.9% (*n* = 505) for day 2. In all samples, the number of embryos per slide did not exceed 110. As in the transfer experiment, the rates of lethality measured via dissection are necessarily underestimates of the true rates because our starting samples are biased against embryos that show abnormalities very early in development.

### Re-feeding and scoring germline regeneration

Starved animals were re-fed by transferring them to 6 cm plates seeded with *E. coli* OP50. The following day, animals were re-transferred to fresh plates to separate them from any larvae that had been mistakenly transferred on the previous day. (Animals were not re-transferred on the first day of re-feeding because starved animals are delicate, and we wanted to reduce the possible trauma of transfer.) After a total of ∼48 h or ∼72 h of re-feeding, animals were examined at 63× magnification.

For each animal, the presence or absence of the following abnormalities was recorded for each germline arm. In the descriptions below, percentages refer to the frequency of each abnormality across all experiments where animals were grown on OP50 prior to starvation (*n* = 5,638); percentages do not sum to 100% because some germlines showed multiple abnormalities. Our classification of these abnormalities as ‘No defect,’ ‘Possible defect,’ or ‘Clear defect’ (Figure 4) reflects the fact that only ‘Clear defects’ generally inhibited embryo production.

Constriction (7.36%): Narrowing of the germline behind the proximal oocytes, as in Figure S4A. Typically, the constriction separated the oogenic and pre-oogenic portions of the germline.

Germline mis-folded (6.51%): Mis-folding of the germline in any number of ways. A severe example is shown in Figure S4C. Less severe examples include: (i) buckling out of the germline just proximal to the spermatheca, and (ii) the distal portion of the germline folding over on itself.

Yolk in the body cavity (8.51%): Yolk in the pseudocoelom of the body half containing the germline in question. When no other abnormalities were present, the amount of yolk was typically small, as in Figure S4D. When yolk was observed in conjunction with necrotic or disorganized germline, yolk often filled the pseudocoelom.

Debris in uterus (7.04%): Debris in the uterus half in question, exclusive of unfertilized oocyte material. In most cases, debris appeared to be the remains of past dead embryos, not yet expelled from the uterus.

Late-stage embryos in utero (2.82%): Late-stage embryos in the uterus half in question. In some cases, uteruses were swollen with embryos.

Germline necrotic or disorganized (3.99%): Germline present but highly disorganized, as in Figure S4E. This category is a heterogeneous catch-all for a variety of severe abnormalities. For most germlines in this category, portions of the germline recognizable as such no longer appeared shrunken (Figure S4E). Germlines in this category were usually not capable of embryo production, as evidenced by the absence of newly fertilized embryos upon re-feeding.

Other (0.66%): Germline absent, similar to a *glp-1* mutant [21] (0.41%); Tumor in the proximal germline (0.14%); Oocytes and pachytene nuclei intermixed, as if oocytes had formed in an incorrect position after re-feeding (0.05%); Germline still shrunken (0.05%).

Normal morphology (71.18%): Germline indistinguishable from the germline of a fed adult that had never been starved.

### *par-2* RNAi

We used the *par-2* (*F58B6.3*) RNAi clone from the Ahringer RNAi feeding library [14], available from Geneservice, Ltd. This clone was grown overnight in 2× YT (MP Biomedicals) containing 50 µg/ml carbenicillin. The culture was concentrated to one third of its original volume and spread onto 10 cm NGM plates containing 50 µg/ml carbenicillin and 1 mM IPTG. L1s were synchronized according to our starvation protocol and plated onto RNAi plates at a density of ∼700 animals per plate. At ‘Mid/early’ L4 and ‘Very late’ L4, food was removed according our starvation protocol. Following the final wash, animals were transferred to glass culture tubes and settled by gravity for ∼20 minutes in ∼15 ml M9. Settling was repeated four additional times. This modification was performed because *par-2* RNAi bacteria are very clumpy and could not be removed by washing alone. Animals were then plated on 10 cm starvation plates at a density of ∼10,000 animals per plate.

Among animals starved from ‘Very late’ L4, embryo lethality induced by *par-2* RNAi was not fully penetrant: Some animals produced at least one viable progeny and died via bagging. (Typically, in such cases, the first one or two embryos per gonad arm were viable, and all subsequent embryos died.) However, many animals failed to bag, thus allowing collection of animals on days 5 and 10 for re-feeding. Re-feeding was performed as described above, with *E. coli* OP50 as the food source.

### Low- versus high-density starvation

Food was removed from ‘Mid/early’ L4 animals, according our starvation protocol above, with two modifications. First, 10 washes in M9 were performed instead of 6. Second, following the final wash, animals were transferred to glass culture tubes and settled by gravity for ∼20 minutes in ∼15 ml M9. Settling was repeated four additional times. These modifications were performed because in initial trials, low-density plates prepared without them often became contaminated during starvation. Next, animals were plated on 10 cm starvation plates at a density of ∼30–50 animals per plate (low-density plates) or ∼10,000 animals per plate (high-density plates). High-density plates were prepared in parallel as a control. During starvation, animals on low-density plates crawled up the walls at high frequencies: By day 10 of starvation, the low-density plates usually contained 5 animals or fewer.

## Supporting Information

Figure S1 **Full dataset for** **Figure 1E–G** **.** (A) Number of embryos or L1s in utero for animals containing at least one embryo or L1. Circles represent individual hermaphrodites. (B) Developmental stages of embryos or L1s in utero. (C) Percent of embryo/L1-containing animals that contain at least one dead embryo. (A–C) Stages of animals at the onset of starvation are indicated in the left-most panel.
(TIF)

Figure S2 **Details of dataset for** **Figure 2** **.** (A) Developmental stages of embryos in tracked animals at the time of transfer (day 3) and upon recovery (day 4). (B) Developmental stages of newly fertilized embryos, at the time of recovery (day 4). (C) Number of new fertilizations per hermaphrodite.
(TIF)

Figure S3 **Additional images of germline shrinkage and germline regeneration.** (A) Germline shrinkage absent in the distal germline. (B) Germ cells bulging out from the rest of the germline. No bulging is observed for the distal germ cells. Inset shows bulging cells at higher magnification. (C) Constriction behind the single oocyte. (D) Constriction with oocyte material on both sides. Inset shows the constriction at higher magnification. (E) Shrinkage in the most proximal pre-oogenic germline only. (F) Germline immediately following fertilization. The uterus contains a 3-fold embryo and an embryo in pseudocleavage. The stages of these embryos indicate that they were ovulated at least 8 h apart. No oocyte material is visible distal to the spermatheca. (G) Germline ∼1 h post-fertilization, as indicated by the most recently fertilized embryo being at the 4-cell stage. Very little oocyte material is visible distal to the spermatheca. (H) Germline ∼8 h post-fertilization, as indicated by the most recently fertilized embryo being at the 2-fold stage. The quantity of oocyte material distal to the spermatheca totals less than half the volume of a mature oocyte. (I) Germline shrinkage in an animal starved at a density of less than 50 animals per 10 cm plate. (J) Germline shrinkage in an animal starved from young adult. The distal germline has regressed away from the vulva, which is located outside the field of view. The germline contains a single oocyte, and hatched L1s are visible in the uterus. (K) Germline regeneration in an animal starved from ‘Very late’ L4 that was grown on *par-2* RNAi prior to starvation. (A–K) Exclusive of insets, all panels are shown at the same magnification; see the magnification bar in (K). Insets are shown at 2× magnification relative to the main panels. When visible, the vulva and distal tip of each germline are marked by a caret and an arrowhead, respectively. In all panels, the age at food removal is indicated to the right of each panel; the day of starvation on which the image was acquired is indicated on each image.
(TIF)

Figure S4 **Additional images of germline regeneration.** (A) Regenerated germline in which the constriction formed during starvation persists. Embryos and unfertilized oocyte material are visible in the uterus. Inset shows the constriction at higher magnification. (B) Relaxed constriction, visible as a furrowing of what might be the sheath cells. Inset shows the relaxed constriction at higher magnification. (C) Germline mis-folding. This mis-folding has not inhibited the production of embryos. (D) Germline categorized as ‘Necrotic or disorganized.’ A portion of the germline near the bend is recognizable and no longer appears shrunken. (E) Spheres of likely yolk in the pseudocoelom of an animal whose germline has otherwise regenerated normally. (A–E) Exclusive of insets, all panels are the shown at the same magnification; see the magnification bar in (E). Insets are shown at 2× magnification relative to the main panels. When visible, the vulva and distal tip of each germline are marked by a caret and an arrowhead, respectively.
(TIF)

Figure S5 **Delay in embryo production for animals starved at low density.** Animals were starved at ‘Mid/early’ L4 and plated, in parallel, at two densities: ∼10,000 per 10 cm plate and less than 50 animals per plate. Surviving animals were collected on days 3, 5, and 10 of starvation and classified according to the criteria described in the legend of Figure 1D. For each population, on each day, *n* = 116–158 animals.
(TIF)
