# Supplementary figures and images for: The Oogenic Germline Starvation Response in C. elegans
Source: PLoS One. 2011 Dec 2;6(12):e28074. doi: 10.1371/journal.pone.0028074 (PMC3229504; doi:10.1371/journal.pone.0028074)

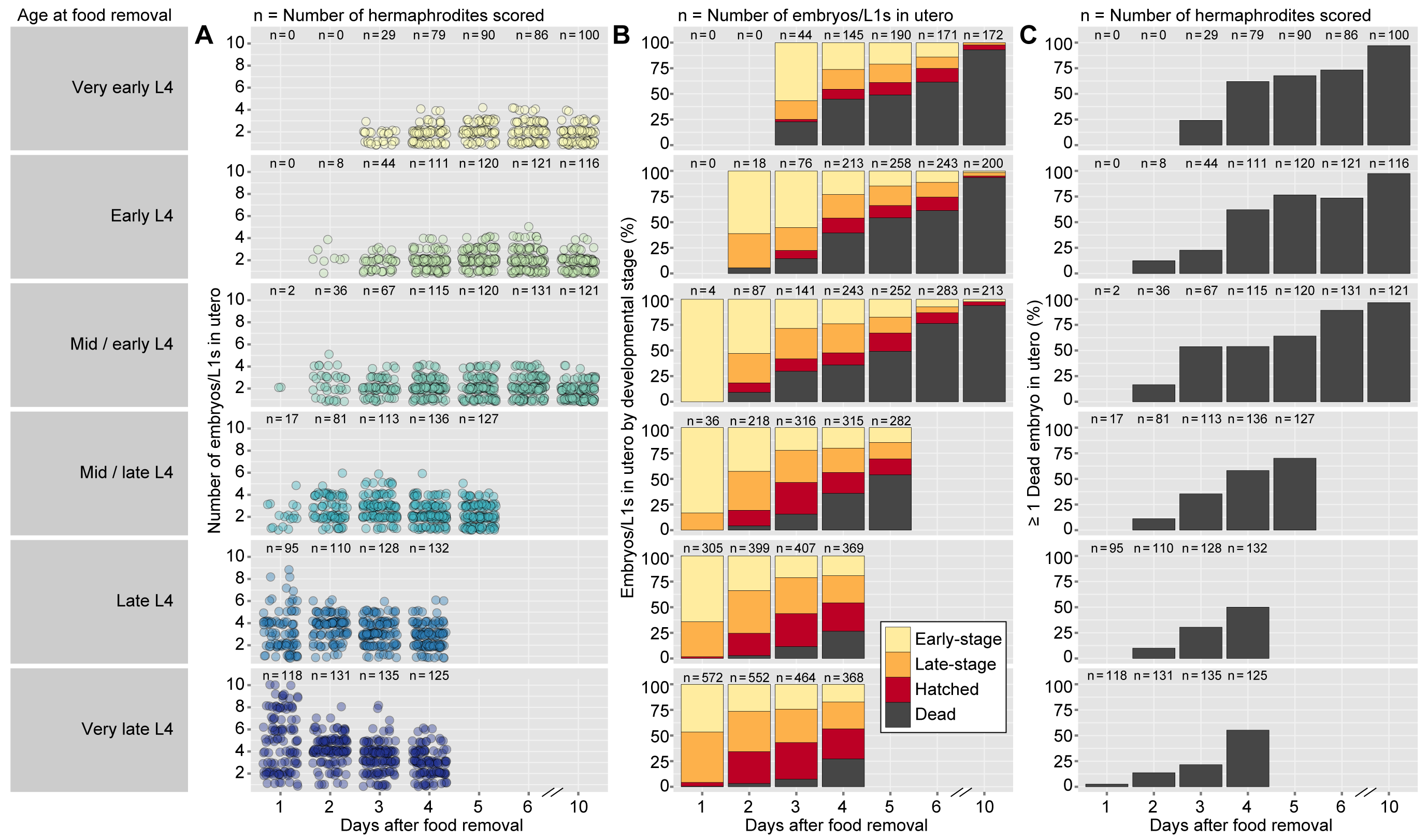

Supplement: Figure S1 — Full dataset for Figure 1E–G . (A) Number of embryos or L1s in utero for animals containing at least one embryo or L1. Circles represent individual hermaphrodites. (B) Developmental stages of embryos or L1s in utero. (C) Percent of embryo/L1-containing animals that contain at least one dead embryo. (A–C) Stages of animals at the onset of starvation are indicated in the left-most panel. (TIF) [file pone.0028074.s001.tif]

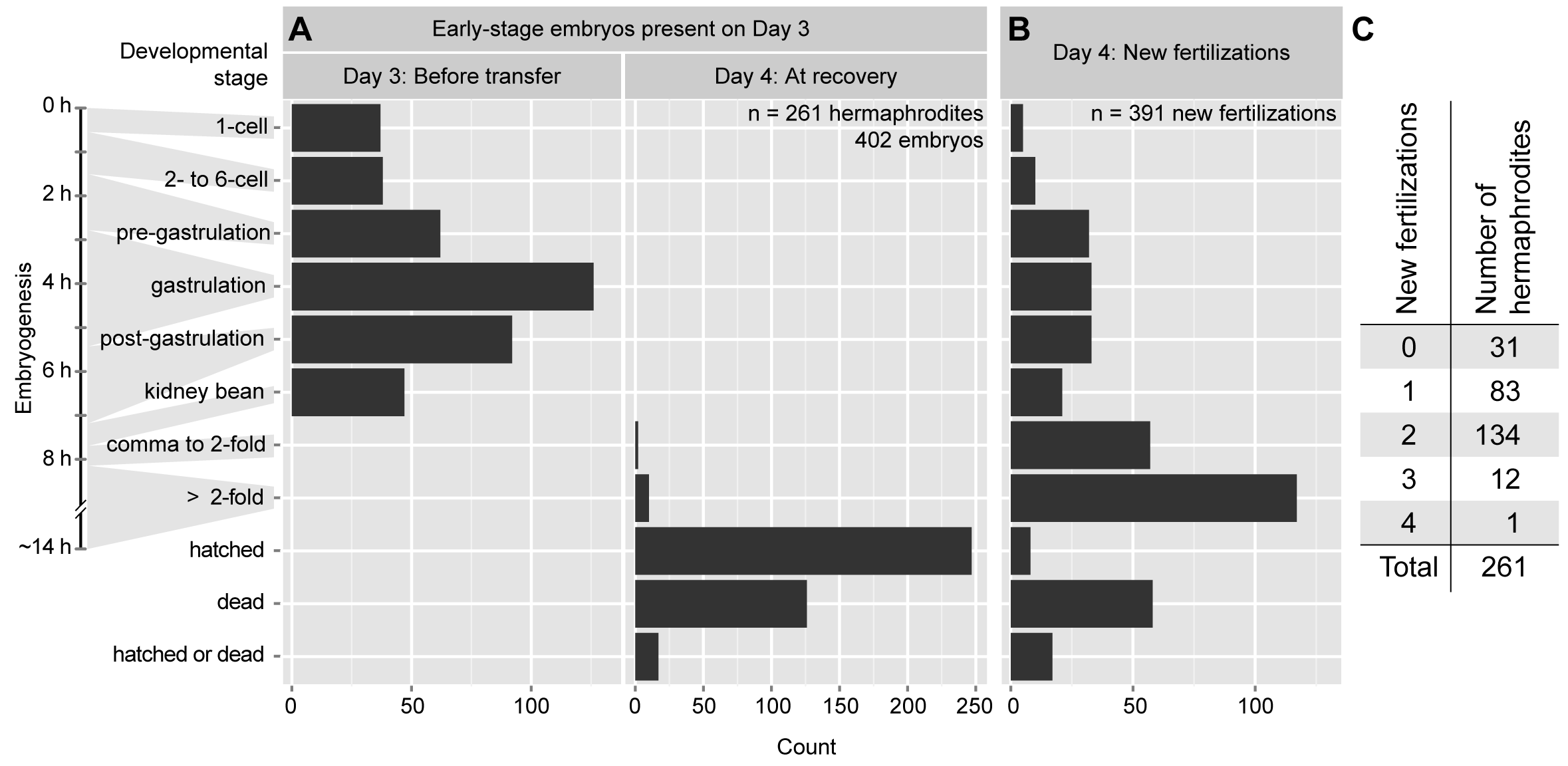

Supplement: Figure S2 — Details of dataset for Figure 2 . (A) Developmental stages of embryos in tracked animals at the time of transfer (day 3) and upon recovery (day 4). (B) Developmental stages of newly fertilized embryos, at the time of recovery (day 4). (C) Number of new fertilizations per hermaphrodite. (TIF) [file pone.0028074.s002.tif]

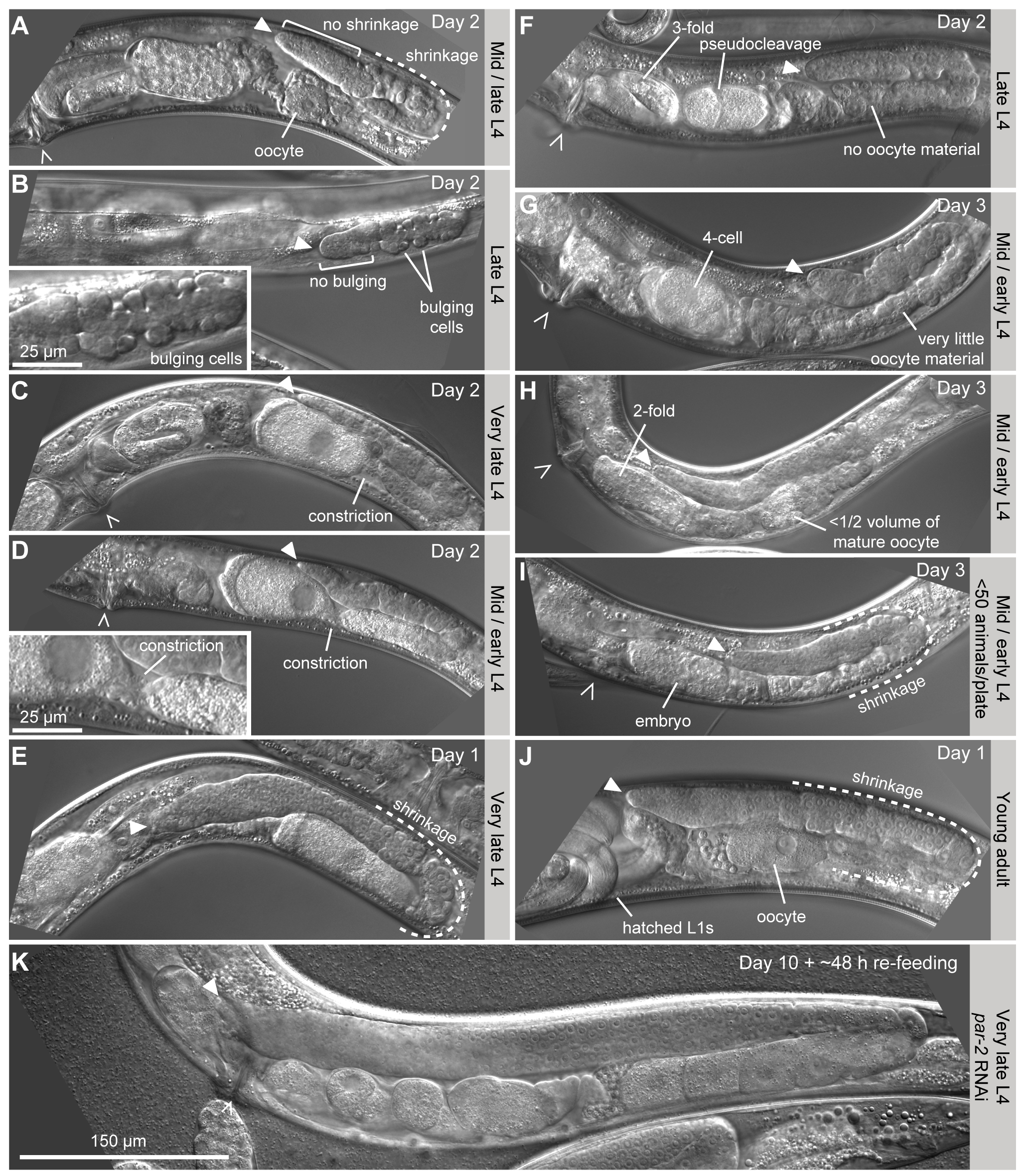

Supplement: Figure S3 — Additional images of germline shrinkage and germline regeneration. (A) Germline shrinkage absent in the distal germline. (B) Germ cells bulging out from the rest of the germline. No bulging is observed for the distal germ cells. Inset shows bulging cells at higher magnification. (C) Constriction behind the single oocyte. (D) Constriction with oocyte material on both sides. Inset shows the constriction at higher magnification. (E) Shrinkage in the most proximal pre-oogenic germline only. (F) Germline immediately following fertilization. The uterus contains a 3-fold embryo and an embryo in pseudocleavage. The stages of these embryos indicate that they were ovulated at least 8 h apart. No oocyte material is visible distal to the spermatheca. (G) Germline ∼1 h post-fertilization, as indicated by the most recently fertilized embryo being at the 4-cell stage. Very little oocyte material is visible distal to the spermatheca. (H) Germline ∼8 h post-fertilization, as indicated by the most recently fertilized embryo being at the 2-fold stage. The quantity of oocyte material distal to the spermatheca totals less than half the volume of a mature oocyte. (I) Germline shrinkage in an animal starved at a density of less than 50 animals per 10 cm plate. (J) Germline shrinkage in an animal starved from young adult. The distal germline has regressed away from the vulva, which is located outside the field of view. The germline contains a single oocyte, and hatched L1s are visible in the uterus. (K) Germline regeneration in an animal starved from ‘Very late’ L4 that was grown on par-2 RNAi prior to starvation. (A–K) Exclusive of insets, all panels are shown at the same magnification; see the magnification bar in (K). Insets are shown at 2× magnification relative to the main panels. When visible, the vulva and distal tip of each germline are marked by a caret and an arrowhead, respectively. In all panels, the age at food removal is indicated to the right of each panel; the day of starva [file pone.0028074.s003.tif]

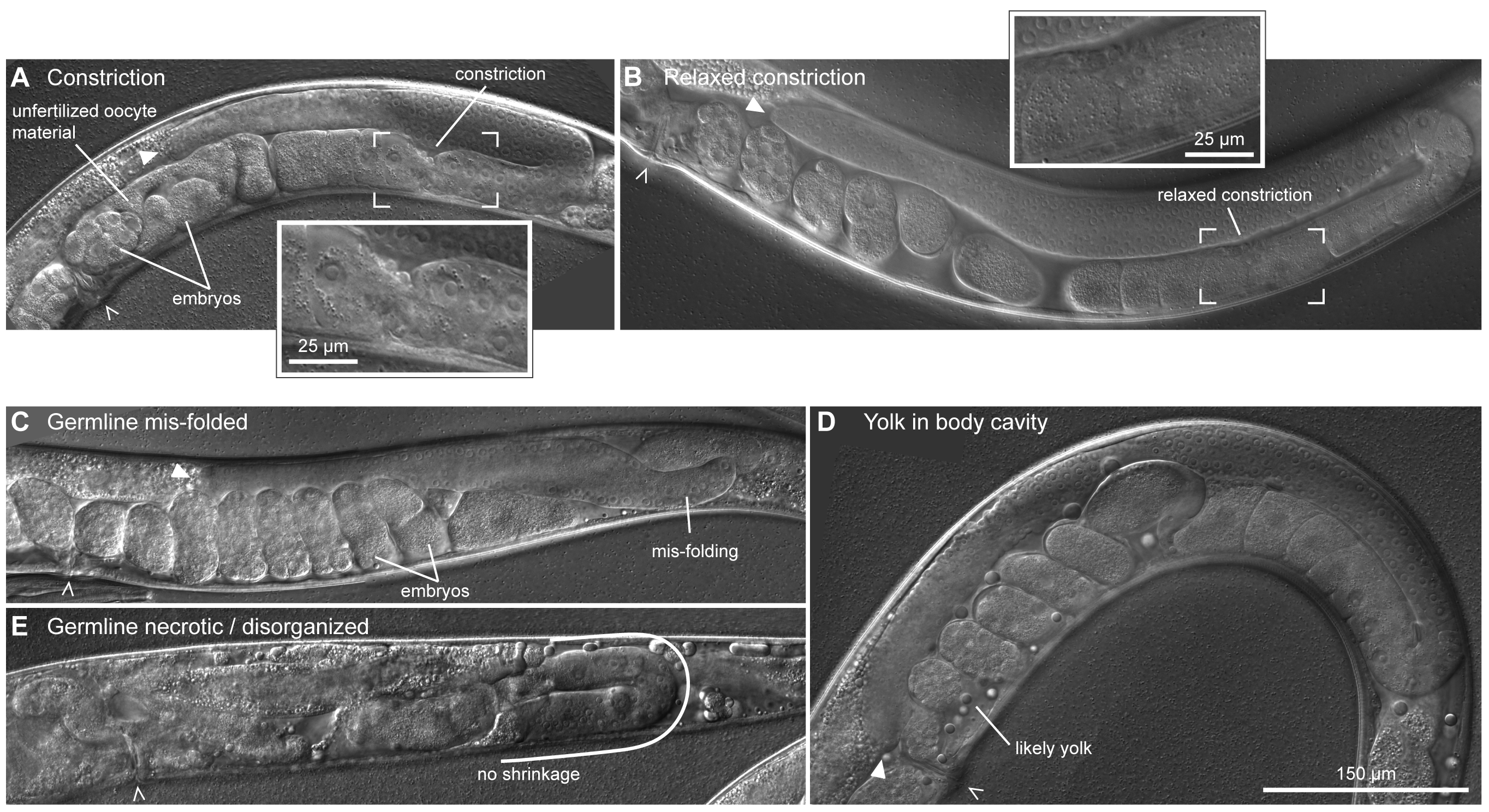

Supplement: Figure S4 — Additional images of germline regeneration. (A) Regenerated germline in which the constriction formed during starvation persists. Embryos and unfertilized oocyte material are visible in the uterus. Inset shows the constriction at higher magnification. (B) Relaxed constriction, visible as a furrowing of what might be the sheath cells. Inset shows the relaxed constriction at higher magnification. (C) Germline mis-folding. This mis-folding has not inhibited the production of embryos. (D) Germline categorized as ‘Necrotic or disorganized.’ A portion of the germline near the bend is recognizable and no longer appears shrunken. (E) Spheres of likely yolk in the pseudocoelom of an animal whose germline has otherwise regenerated normally. (A–E) Exclusive of insets, all panels are the shown at the same magnification; see the magnification bar in (E). Insets are shown at 2× magnification relative to the main panels. When visible, the vulva and distal tip of each germline are marked by a caret and an arrowhead, respectively. (TIF) [file pone.0028074.s004.tif]

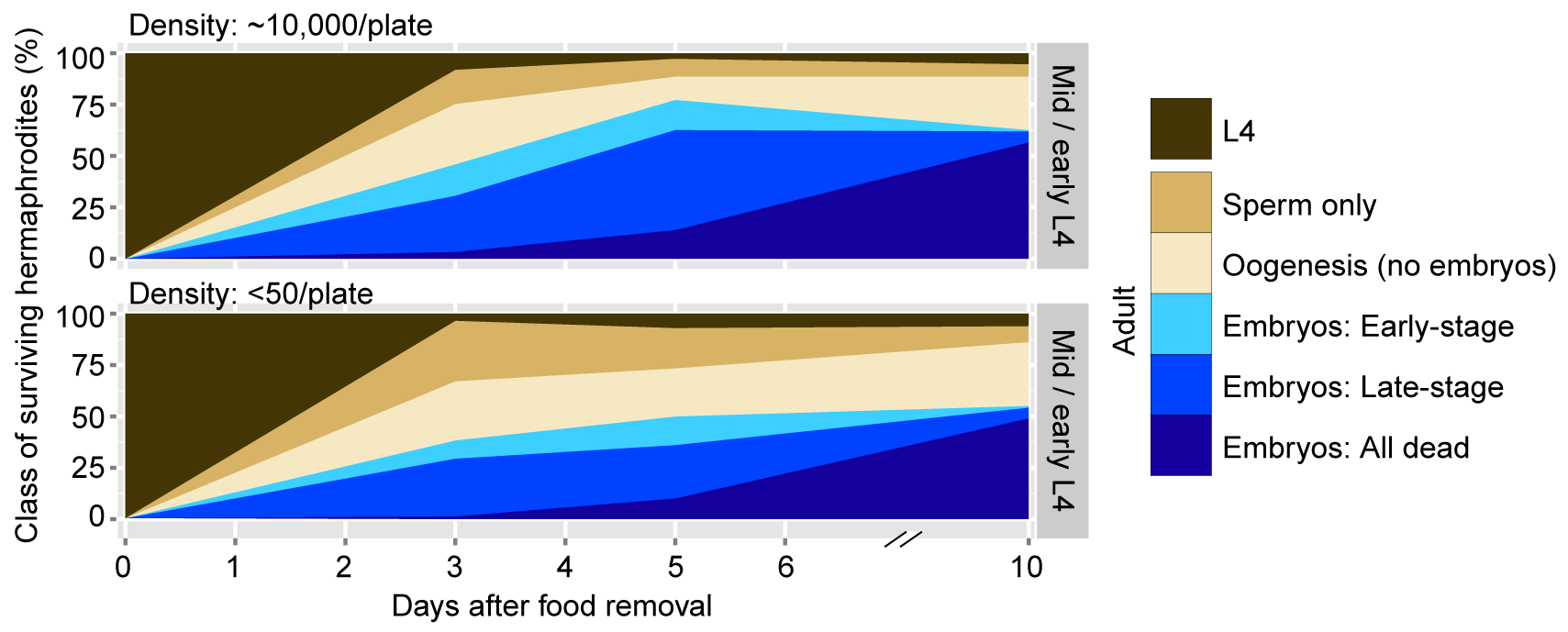

Supplement: Figure S5 — Delay in embryo production for animals starved at low density. Animals were starved at ‘Mid/early’ L4 and plated, in parallel, at two densities: ∼10,000 per 10 cm plate and less than 50 animals per plate. Surviving animals were collected on days 3, 5, and 10 of starvation and classified according to the criteria described in the legend of Figure 1D. For each population, on each day, n = 116–158 animals. (TIF) [file pone.0028074.s005.tif]
